# The Effect of Preoperative Oral Carbohydrate or Oral Rehydration Solution on Postoperative Quality of Recovery: A Randomized, Controlled Clinical Trial

**DOI:** 10.1371/journal.pone.0133309

**Published:** 2015-08-28

**Authors:** Ayako Asakura, Takahiro Mihara, Takahisa Goto

**Affiliations:** 1 Department of Anesthesiology and Critical Care Medicine, Yokohama City University Graduate School of Medicine, Yokohama, Japan; 2 Department of Anesthesiology, Kanagawa Children’s Medical Center, Yokohama, Japan; The Chinese University of Hong Kong, HONG KONG

## Abstract

**Background:**

Numerous studies have demonstrated the beneficial effects of preoperative administration of oral carbohydrate (CHO) or oral rehydration solution (ORS). However, the effects of preoperative CHO or ORS on postoperative quality of recovery after anesthesia remain unclear. Consequently, the purpose of the current study was to evaluate the effect of preoperative CHO or ORS on patient recovery, using the Quality of Recovery 40 questionnaire (QoR-40).

**Methods:**

This prospective, randomized, controlled clinical trial included American Society of Anesthesiologists (ASA) physical status 1 and 2 adult patients, who were scheduled to undergo a surgical procedure of body surface. Subjects were randomized to one of the three groups: 1) preoperative CHO group, 2) preoperative ORS group, and 3) control group. The primary outcome was the global QoR-40 administered 24 h after surgery. Intraoperative use of vasopressor, intraoperative body temperature changes, and postoperative nausea and vomiting (PONV) were also evaluated.

**Results:**

We studied 134 subjects. The median [interquartile range (IQR)] global QoR-40 scores 24 h after the surgery were 187 [177–197], 186 [171–200], and 184 [171–198] for the CHO, ORS, and control groups, respectively (*p* = 0.916). No significant differences existed between the groups regarding intraoperative vasopressor use during the surgery (*p* = 0.475).

**Conclusions:**

Results of the current study indicated that the preoperative administration of either CHO or ORS did not improve the quality of recovery in patients undergoing minimally invasive body surface surgery.

**Trial Registration:**

www.umin.ac.jp UMIN000009388 https://upload.umin.ac.jp/cgi-open-bin/ctr/ctr.cgi?function=brows&action=brows&type=summary&recptno=R000011029&language=E

## Introduction

Enhanced postoperative recovery programs have become widely accepted, and allowing the unrestricted intake of clear liquids until 2 h before anesthesia has been adopted as a standard practice [1].

A number of prior studies have examined the effects of preoperative administration of oral carbohydrate (CHO), and have resulted in reports of beneficial effects of CHO on postoperative insulin resistance [2,3], muscle function [4], gut function [5], immunodepression [6], and preoperative discomfort [7]. As well, some prior research emphasized the supplementation of fluid and electrolytes through the administration of preoperative oral rehydration solution (ORS) [8]. However, the effects of CHO or ORS administration on important clinical endpoints such as postoperative quality of recovery (QoR) following anesthesia remain uncertain [9,10]. Additionally, no prior research has endeavored to compare the effects of preoperative CHO or ORS on postoperative QoR. Accordingly, we considered it important to clarify whether CHO or ORS affected postoperative QoR, which solution was easier to apply in clinical practice, and which would be beneficial for a large number of patients.

As we considered that in invasive surgery the stress response from the surgical trauma is too large to clarify the effects of preoperative administration, we decided to assess the effects in minor surgery.

The aim of the current study was to investigate the effects of preoperative administration of CHO or ORS on patient recovery from minor surgery by using the Quality of Recovery 40 (QoR-40) questionnaire.

## Materials and Methods

### Study Subjects

The study was a prospective, controlled, parallel-group clinical trial with equal randomization conducted at the Yokohama City University Hospital in Yokohama, Japan. The study was approved by the Ethics Committee of Yokohama City University Hospital on December 1^st^, 2011 with approved number B111110025, and written informed consent was obtained from all study participants. The patients were recruited from January 5^th^, 2012 to March 21^st^, 2013, and were followed-up from January 5^th^, 2012 to March 23^rd^, 2013. The clinical trial registration for this study can be found at www.umin.ac.jp under registration number UMIN000009388. There was a delay in registering this study by misunderstanding.

Eligible participants were ASA physical status 1 and 2 adults, age 20 to 79 years, who were scheduled to undergo a surgical procedure of body surface. In the original study protocol, the subjects were patients who were scheduled to undergo a transperineal prostate brachytherapy or lymphaticovenular anastomosis, but were broadened as written above after the Ethics Committee approved our intention of the change. Patients with impaired gastrointestinal motility, poor comprehension of Japanese, or with psychiatric disorders were excluded from enrolment.

Patients were randomly assigned to one of three preoperative treatment groups. Group 1 received 250ml of preoperative CHO (Arginaid Water ™, 18% carbohydrates, Nestle Health Science, Tokyo, Japan) between 6.00–6:30 a.m. on the morning of surgery. This is because 250ml of Arginaid Water are approved as a meal. Group 2 received 1000ml of preoperative ORS (OS-1 ™, 2.5% carbohydrate, Otsuka Pharmaceutical, Tokushima, Japan) starting 20.00 p.m. on the night before surgery and up to 2 h prior to anesthesia induction. This is based on the volume for rehydrating 12h of dehydration in healthy patients. Group 3 served as the control group, did not receive any preoperative CHO or ORS, and were fasted starting at midnight on the day of surgical procedure. The composition of the test solutions are detailed in Table 1 and as previously mentioned, the amount were selected based on clinical use.

**Table 1:** Composition of Each Test Fluid.

	CHO (Arginaid Water)	ORS (OS-1)
Energy (kcal/ml)	0.8	0.1
Carbohydrate (%)	18	2.5
Glucose (%)		1.8
Sodium (mEq/L)	0	50
Potassium (mEq/L)	_	20
Magnesium (mEq/L)	_	2
Lactate (mEq/L)	_	31
Chloride (mEq/L)	_	50
Phosphorus (mEq/L)	0.18	2
Zinc (%)	0.008	_
Copper (%)	0.0008	_
L-Arginine (%)	2	_
Osmolality (mOsm/L)	545	270

Randomization of the study participants was performed after written informed consent was obtained from the study participants, by a lottery method using sealed opaque lot. Although patients and anesthesiologists were not blinded to the patient’s group allocation, a data analyst was blinded.

### Anesthesia and surgical management

None of the study subjects received any premedication. Fentanyl 1–2 μg kg^-1^ and propofol 1–2 mg kg^-1^ were administered for inducing anesthesia. Rocuronium 0.6 mg kg^-1^ was administered to facilitate tracheal intubation or laryngeal mask airway insertion. Anesthesia maintenance was achieved using end-tidal sevoflurane 1.0–1.5% and remifentanil 0.05–0.20 μg kg^-1^ min^-1^, titrated to keep the mean blood pressure within 20% of the baseline. Additional fentanyl and rocuronium were administered during the procedure, if necessary. During maintenance, the patients received a mixture of air and oxygen (O_2_) to keep F_I_O_2_ between 0.4 and 0.6. The patients received 50 mg flurbiprofen 15 min before the end of the procedure, if needed. Neuromuscular blocks were reversed by the administration of neostigmine and atropine.

All surgical procedures performed were minimally invasive and were associated with either no postoperative pain or pain that was easily managed by administration of non-steroidal anti-inflammatory drugs (NSAIDs).

### Data Collection

Twenty-four hours after the conclusion of the surgical procedure, the subjects were asked to complete the Japanese version of QoR-40 questionnaire [11]. The QoR-40 is a global measure of quality of recovery that incorporates five dimensions of health including patient support, comfort, emotions, physical independence, and pain, each graded on a 5-point Likert scale. The QoR-40 scores range from 40 (extremely poor quality of recovery) to 200 (excellent quality of recovery). Additional perioperative data collected included the subject’s age, height, weight, ASA physical status, preoperative QoR-40 scores, duration of the anaesthesia, total intravenous (IV) fluids delivered, incidence of aspiration, intraoperative use of a vasopressor, intraoperative changes in body temperature, and postoperative nausea and vomiting (PONV). 36-Item Short-Form Health Survey (SF-36 ™) of 1 and 3months after the surgey were also planned in the original protocol to evaluate the postoperative quality of life, but we decided to specialize only in the postoperative quality of recovery, so only the QoR-40 questionnaire were used.

### Statistical Analysis

The primary outcome of the study was the global QoR-40 score. A sample size of 42 per group was estimated to achieve 80% power with a significance level of 0.05 to detect a 10-point difference in the aggregated QoR-40 score for the 3 study groups to be compared assuming an overall standard deviation of 14, similar to what was observed in a previous investigation [12]. A 10-point difference represents a clinically relevant improvement in quality of recovery based on previously reported values on the mean and range of the QoR-40 score in patients after anaesthesia and surgery [13]. To account for the loss of some study participants, 150 subjects were recruited. The sample size calculation was made using the R statistical software package version 2.13.0 (R Foundation for Statistical Computing, Vienna, Austria). In the original protocol, 250 subjects were planned based on the previous study [7], however following the result of calculation, 150 subjects were recruited.

The analyses were performed on data from the intention-to-treat population, defined as all randomly assigned patients except for those who had no complete QoR-40. In the per-protocol analyses, patients with one or more major protocol violations were excluded.

The Kolmogorov-Smirnov test was used to test the hypothesis of normal distribution. Normally distributed data were reported as mean ± [standard deviation (SD)] and were analyzed using one-way analysis of variance (ANOVA). Non-normally distributed data are reported as median [interquartile range (IQR)] and were analyzed using the Kruskal-Wallis test. When there was a significant difference, a post hoc analysis with Bonferroni correction was planned to be performed, however with no significant differences in Kruskal-Wallis test, we did not perform the paired comparison. Categorical variables were compared using the Fisher’s exact test. All statistical analyses were performed using the modified R software programs version 1.11 [14], and *p*-values of <0.050 were considered statistically significant.

## Results

**Fig 1:**
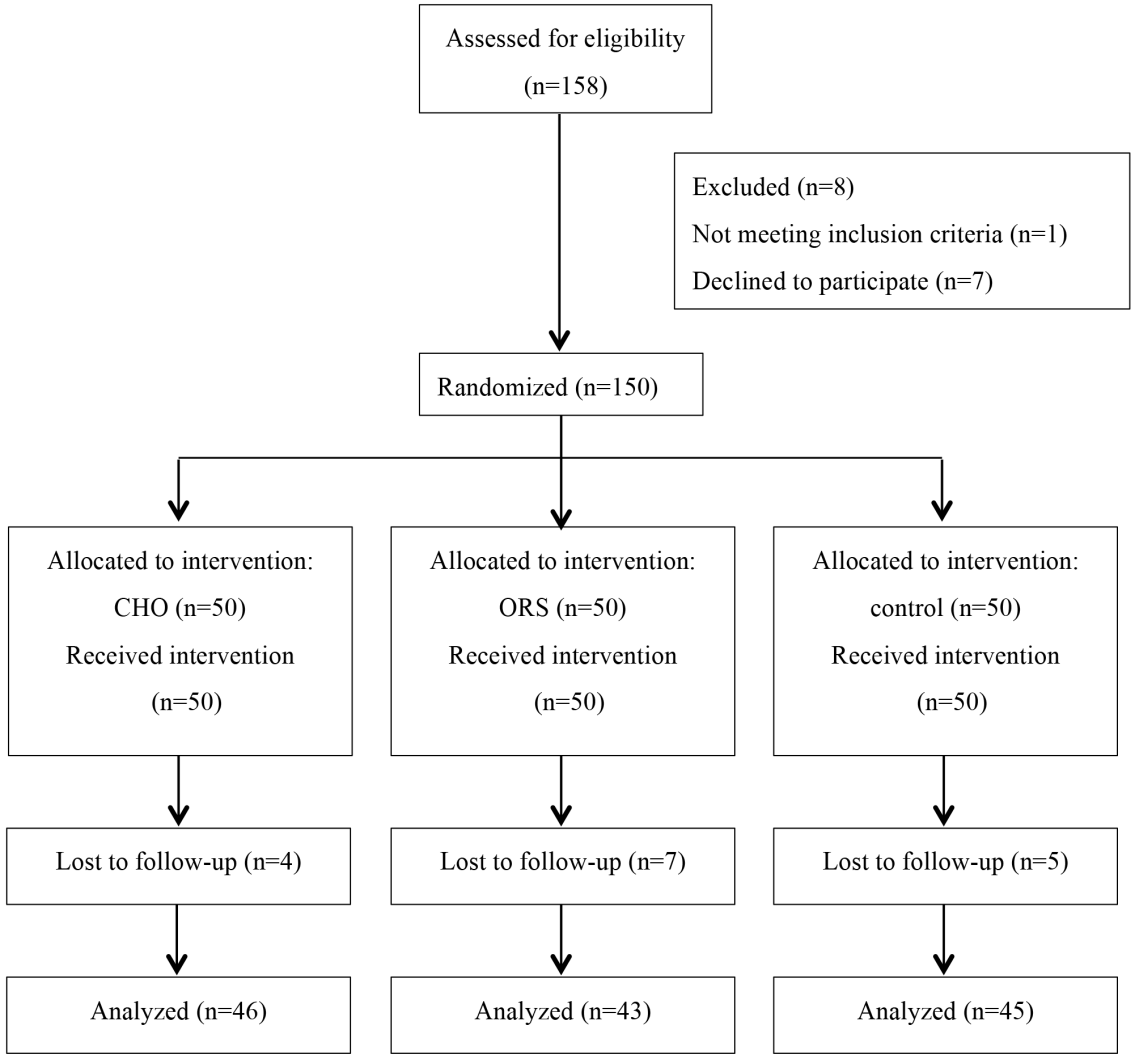
Consort flow study diagram.

The details of the conduct of the study are shown in Fig 1. Out of the 150 subjects originally enrolled and randomized into treatment groups, 134 subjects completed the study. Sixteen patients were lost to follow-up: three patients failed to complete the questionnaire, and for the remaining 13 patients, we were unable to collect the questionnaire. We could not find any statistically significant differences among the groups in the patients’ baseline characteristics, preexisting medical conditions, and surgical factors (Table 2). In the current study, two-thirds of the surgical cases involved patients undergoing transperineal prostate brachytherapy, which resulted in a patient population comprising 70% men (Table 2).

**Table 2:** Subjects characteristics and clinical details. Data presented as mean ± SD, median (interquartile range), or number (%).

	CHO (*n* = 46)	ORS (*n* = 43)	Control (*n* = 45)	*p* value
Age (yr)	63.4 ±13.6	64.4 ± 9.7	64.5 ± 10.4	0.877
Sex				0.891
M	31 (67)	31 (72)	32 (71)	
F	15 (33)	12 (28)	13 (29)	
Height (m)	1.62 ± 0.06	1.63 ± 0.08	1.63 ± 0.07	0.456
Weight (kg)	61.2 ± 9.3	64.1 ± 8.9	62.9 ± 10.1	0.345
ASA physical				0.037
Ⅰ	17 (37)	7 (16)	16 (36)	
Ⅱ	29 (63)	36 (84)	29 (64)	
Duration of anesthesia (min)	142 (120–170)	150 (121–218)	137 (118–309)	0.804
Duration of surgery (min)	59.5 (39.3–113)	64.0 (49.5–144)	59.0 (43.0–140)	0.764
Intravenous infusion (ml)	800 (600–1000)	800 (700–1100)	800 (675–1275)	0.740
Type of surgery				0.736
Urological	29 (63)	27 (63)	31 (69)	
Gynecological	5 (11)	2 (4.6)	1 (2.2)	
Plastics	8 (17)	9 (21)	11 (24)	
ENT	1 (2.2)	2 (4.6)	1 (2.2)	
Orthopedics	1 (2.2)	2 (4.6)	0 (0)	
General	2 (4.3)	1 (2.3)	1 (2.2)	
Length of hospital stay (day)	3 (2–3)	3 (2–3)	3 (2–6)	0.794
Hypertension	17 (37)	16 (37)	20 (44)	0.557
Diabetes	4 (8.7)	5 (12)	7 (16)	0.600
Smoking history	19 (41)	23 (54)	18 (40)	0.382
Global QoR-40 score	196 (191–198)	195 (185–200)	197 (189.5–200)	0.655

ENT, ear, nose, or throat

**Fig 2:**
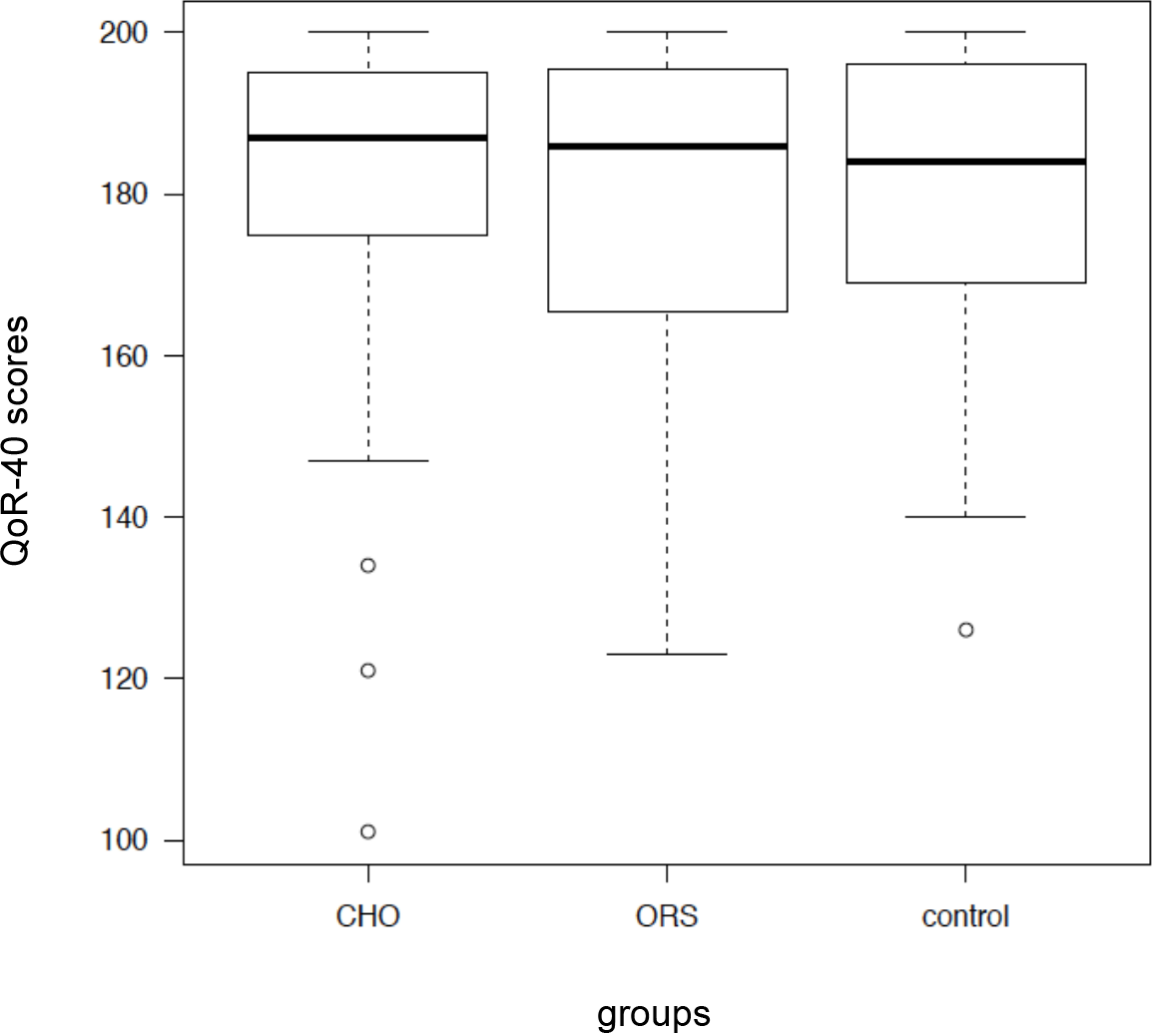
Box plot of the global QoR-40 scores 24 h after surgery. Median values shown as solid line within box of 25 and 75^th^ percentile values. Whiskers represent 10 and 90^th^ percentile values. No significant differences were detected among the groups.

The median (IQR) of the global QoR-40 scores 24 h after surgery were 187 (177–197), 186 (171–200), and 184 (171–198) for the CHO, ORS, and control groups, respectively (Fig 2). Further, no statistical and clinical differences (*p* = 0.916) were detected between the global QoR-40 scores from the different treatment groups (Table 3). Likewise, there were no differences in each dimensions of the QoR-40 (Table 3).

**Table 3:** Global and five-dimensional postoperative quality of recovery scores. Data presented as median (interquartile range).

	CHO (*n* = 46)	ORS (*n* = 43)	Control (*n* = 45)	*p* value
QoR-40 dimensions				
Emotional state	43 (39–45)	43 (38–45)	42 (38.5–45)	0.984
Physical comfort	56 (52–59)	56 (51–59)	56 (48–59)	0.822
Patient support	35 (33–35)	35 (33–35)	35 (31–35)	0.922
Physical independence	24 (19–25)	23 (16–25)	24 (18–25)	0.896
Pain	33 (29–34)	32 (27–33)	32 (29–34)	0.486
Global QoR-40	187 (175–195)	186 (165–196)	184 (168.5–196)	0.916

One patient from the CHO group and six patients from the ORS group were not able to drink the prescribed amount of solution preoperatively. In addition, dexamethasone and/or droperidol had to be administered to three patients from the CHO group, three patients from the ORS group, and one patient from the control group, who complained of nausea or vomiting in the operating room. Accordingly, in the per-protocol analyses, data from these patients (*n* = 7) were excluded. The median (IQR) global QoR-40 scores were 187.5 (175.5–195), 188 (165.75–196), and 184.5 (168.75–196) for the CHO, ORS, and control groups, respectively. The results had no significant difference with the intention-to-treat analyses, and showed no statistical and clinical difference (*p* = 0.831).

The requirement for use of a vasopressor agent (ephedrine or phenylephrine) during the surgery did not differ significantly among the groups both in the intention-to-treat (*p* = 0.475) and per-protocol analyses (*p* = 0.344). Moreover, the incidence of PONV did not differ significantly among the treatment groups in either the intention-to-treat (*p* = 1.000) or per-protocol analyses (*p* = 1.000). (Table 4).

**Table 4:** Incidence of intraoperative vasopressor use and PONV. Data presented as number (%).

ITT	CHO (*n* = 46)	ORS (*n* = 43)	Control (*n* = 45)	*p* value
Required vasopressor	38 (83)	34 (79)	40 (89)	0.475
PONV	4 (8.7)	4 (9.3)	4 (8.9)	1.000
PP	CHO (*n* = 42)	ORS (*n* = 34)	Control (*n* = 44)	*p* value
Required vasopressor	34 (81)	28 (82)	40 (91)	0.344
PONV	2 (4.8)	3 (8.8)	2 (4.5)	1.000

**Fig 3:**
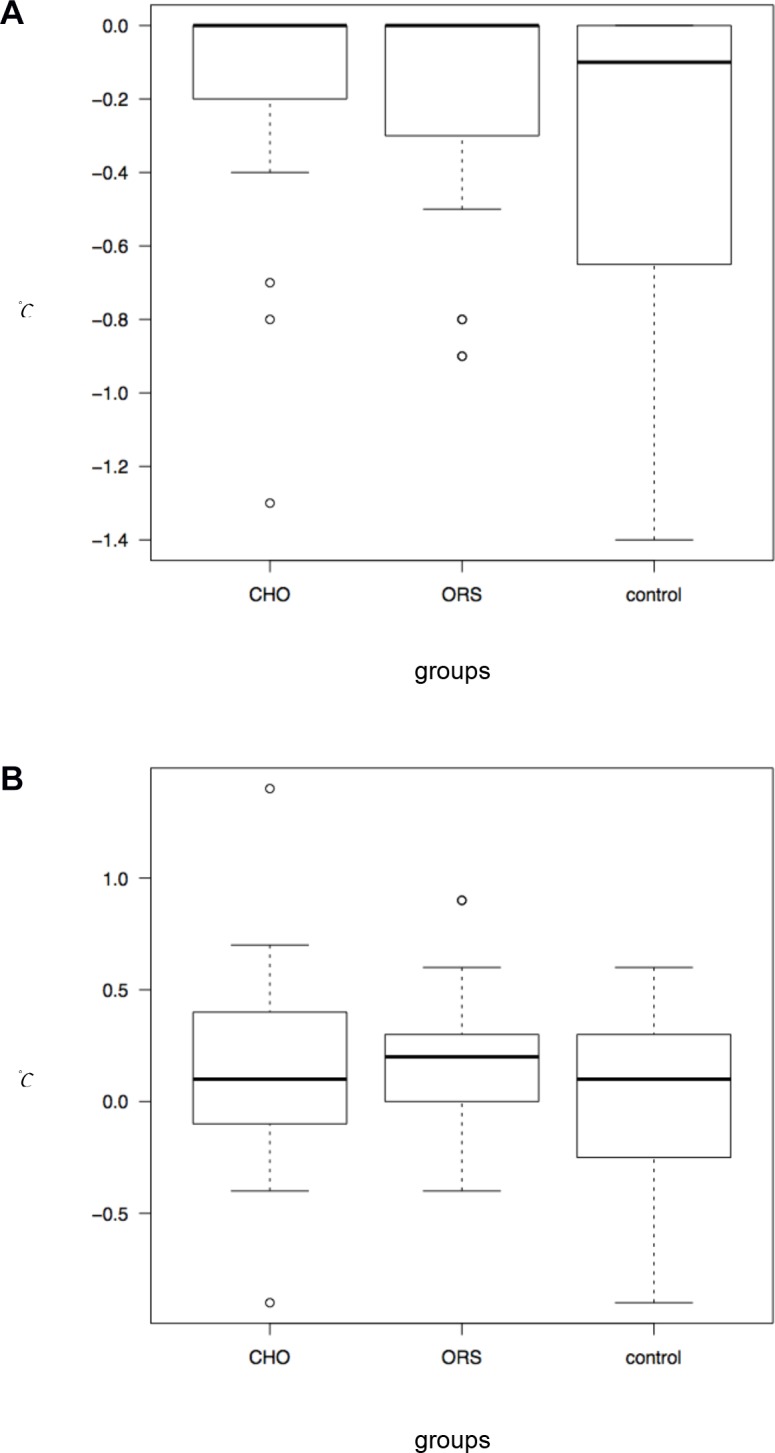
A. Box plot of the maximum decrease of temperatures from the baseline. No significant differences were detected among the groups, but the maximum decreases tended to be smaller in the CHO and ORS groups than in the control group. **B. Box plot of the final temperature changes at the end of surgery.** No significant differences were detected among the groups. No patients in either the CHO or ORS groups aspirated during any of the procedures.

Baseline body temperatures were measured immediately after induction, and the maximum decreases from the baseline, as well as the final temperature changes at the end of surgery relative to the baseline, were assessed. The maximum decreases (IQR) from the baseline were 0.0 (-0.3–0), 0.0(-0.3–0), and -0.1(-0.6–0) in the CHO, ORS, and control groups, respectively (*p* = 0.136) (Fig 3A), while the final temperature changes (IQR) compared to the baseline were 0.1 (-0.1–0.4), 0.2 (0–0.4), and 0.1(-0.2–0.3) in the CHO, ORS, and control groups, respectively (*p* = 0.165) (Fig 3B). In the per-protocol analyses, no significant differences were detected among the groups (*p* = 0.069 and *p* = 0.158). However, the maximum decreases tended to be smaller in the CHO and ORS groups than in the control group.

## Discussion

The median (IQR) global QoR-40 score 24 h after the surgery indicated that no statistical or clinical differences existed among the preoperative CHO, preoperative ORS, and control groups. Similarly, there were no differences among the groups in the requirement to use a vasopressor during surgery. Similarly, there were no differences in temperature changes.

Our sample size was determined to detect a clinically important difference (at least 10) in QoR40 score. Therefore, based on our negative results, we can conlclude that in minor surgery, the preoperative intake of either CHO or ORS had no clinically relevant effect on QoR improvement. Two possible explanations for the results of the study could be considered. One explanation is that, the QoR-40 scores in the control group were exceptionally high, which made it difficult to achieve a clinically relevant difference when compared to the QoR-40 scores in the CHO or ORS groups. Results of a systematic review of the QoR-40 indicated that, the mean QoR-40 score for patients without postoperative complications was 170 [15]. However, in this meta-analysis, major surgical procedures such as cardiac surgery, neurosurgery, and general surgery were included. Compared to the prior analyses, the scores we obtained in the control group were much higher, most likely because the surgeries included in our analyses were less invasive. A second explanation is that despite the beneficial effects of preoperative CHO and/or ORS have been reported, the effects when used prior to minor surgeries could be much less. Previously published studies have mostly included abdominal surgery, and there are no reports of preoperative CHO and/or ORS administration in minor surgeries. Thus, an assumption could be made that due to the minimally invasive nature of the surgeries included in the study, the patients recovered well independent of the preoperative administration of CHO and/or ORS.

Although the administration of either CHO or ORS was thought to restore circulatory volume and potentially result in a reduced reliance on vasopressor use during the surgery when compared to the control group, there was no significant difference among the treatment groups. However, there are reports that preoperative fasting does not induce significant hypovolemia, and that preoperative ORS does not affect the magnitude of hypotension [16–18], which was in accord with our current results. Likewise, as was previously mentioned, two thirds of the cases in the current study included patients undergoing the transperineal brachytherapy for the treatment of prostate cancer. Despite the minimal invasiveness of the procedure, immobilization with muscle relaxant and strong analgesia are required. Thus, the assumption was made that, blood pressure inevitably dropped, and the use of a vasopressor was required in all the groups.

In body temperature assessments, we were not able to achieve any positive results, but showed the propensity to maintain temperatures in the per-protocol analyses. Preoperative CHO loading has been shown to attenuate the development of hypothermia during general anaesthesia in rats [19]. However, in this study, no surgical procedures had been performed, which may not accurately reflect a clinical practice. In a clinical study that investigated the effects of preoperative CHO intake on thermoregulation, significant decreases in postoperative shivering were reported in the CHO group compared to the control group, but no differences were apparent in intraoperative temperatures [20]. Preoperative intake of CHO or ORS may have some effect on the prevention of intraoperative hypothermia, but the effects are probably too minor to cause any significant changes in temperature. In our study, there was only one case of postoperative shivering in the control group; thus, we cannot discuss whether preoperative administration of CHO had any effect on prevention of intraoperative hypothermia.

There are limitations to our study. First, there was imbalance in the type of surgeries performed, which led to a predominance of male patients, and elderly men in particular. Patient sex is an independent factor that influences post-surgical recovery responses, and prior reports have indicated that QoR tends to be better in men than in women [21]. Therefore, the QoR-40 score may be lower if the number of women included in the study is disproportionately high, and the results could be different. Moreover, whether the patient age has any influence in QoR is unknown, but the potential exists that results may be different in younger patients. Second, the patients were aware of the group to which they had been allocated. Thus, concerns existed that there may have been some psychological effects on QoR-40 when patients who wished to receive either CHO or ORS were allocated to the control group. However, there were no differences in each QoR-40 dimensions, including emotional state, which indicated that the actual impact of our concerns was minimal. Third, there was no placebo group (i.e., preoperative clear fluid without CHO load) in our study, which would potentially make it diffcult to interpret our results, especially if the results was positive. However, we considered that the conclusions derived from our negative results would not be affected by lack of a placebo group. This is because the effect of placebo fluid on QoR was asssumed to be between that of the fasted group and the CHO/ORS groups, and we have shown that the effect of these groups on QoR were not different.

In conclusion, the preoperative administration of either CHO or ORS had little impact on improving the quality of recovery in current study, with the surgical proedures performed were less invasive, and the patient population was largely comprised of elderly men. Accordingly, further studies are needed to confirm if the results would differ for more invasive surgeries, or in women and younger patients.

## Supporting Information

S1 CONSORT Checklist CONSORT Checklist. (DOC)

S1 Protocol Protocol, original. (DOC)

S2 Protocol Protocol, English version. (DOCX)

S1 Supporting Information Application for the Ethics Committee, original. (DOC)

S2 Supporting Information Application for the Ethics Committee, English version. (DOCX)
