# Supplementary material for: The Effect of Preoperative Oral Carbohydrate or Oral Rehydration Solution on Postoperative Quality of Recovery: A Randomized, Controlled Clinical Trial
Source: PLoS One. 2015 Aug 28;10(8):e0133309. doi: 10.1371/journal.pone.0133309 (PMC4552663; doi:10.1371/journal.pone.0133309)
Supplement: S1 Protocol — (DOC) [file pone.0133309.s002.doc]

【2011年2月1日改訂】

様式第２号

平成23年8月8日

臨床研究実施概要書

【研究責任者】　麻酔科　　朝倉　彩子

| １　開発・研究名 | 術前禁飲水、術前経口補水、術前炭水化物補水における麻酔覚醒の質　およびQOLの比較 |
| --- | --- |
| ２　研究責任者 | 所属　麻酔科　　　　職名 　 助教　　　　 氏名　朝倉　彩子 |
| ３　分担研究者 | 所属 麻酔科 　　職名　　教授　　　 氏名　後藤　隆久  手術部　　　　　　 　准教授 　　 宮下　徹也  　　　麻酔科 　　　　　　講師 　　　 渡邊　至 麻酔科 　　　　　　講師　　 　　　 伊奈川　岳  麻酔科 　　　　　　助教 　　　 近藤　竜也  麻酔科 　　　　　　助教 　　　 川上　裕理  麻酔科 　　　　　　助教 　　　 佐藤　仁  　　　麻酔科　　　　　 　　助教　　　　　　　 刈谷　隆之  麻酔科 　　　　　　助教 　　　 山口　嘉一  麻酔科 　　　　　　助教 　　　 横瀬　真志  麻酔科 　　　　　　助教 　　　 寺端　昭博  麻酔科 　　　　　　助教 　　　 坪井　さやか  麻酔科 　　　　　指導診療医 　　　 夏川　恭子  麻酔科 　　　 　指導診療医 　 佐藤　大樹  　　 麻酔科　　　　　　 指導診療医　　　　　 迫田　厚志 |
| ４　個人情報管理者 | 所属 麻酔科 　職名　　講師 氏名　水野　祐介 |
| ５　研究の背景・意義  （この研究がなされるに至った過程や期待される成果） | 近年、手術患者の回復力強化のために、麻酔導入2時間前まで飲水可能とし、経口補水液もしくは炭水化物含有飲料水を摂取することが推奨されてきている。術前に経口補水することで、周術期不快感が減少されることが認められている。このため、従来の禁飲水群に比べ、術前補水液摂取群では麻酔覚醒の質が高いことが仮定される。 |
| ６　目　的 | 小線源埋め込み術およびリンパ管静脈吻合において、従来通りの術前禁飲水群、術前経口補水群、術前炭水化物補水群の3群で、術後麻酔覚醒の質や術後QOLを比較すること。 |
| ７　対　象 | 対象（性、年齢、健常人か特定の疾病の患者か、適格基準、除外基準を含む）  小線源埋め込み術およびリンパ管静脈吻合が予定されている20-79歳のASA PS1, 2の患者  消化管機能低下患者、胃食道逆流患者、気道確保困難が予想される、  日本語が理解できないもしくは精神/中枢神経系疾患の患者は除外  対象症例数（算出の根拠を含む）  250名。2001年Anesthesia and Analgesia(麻酔科で2番目にIFの高い学術雑誌)に出た、術前禁飲水群と術前経口炭水化物補水群とを比較した論文では250例で周術期不快感の差が検出されている。 |
| ８　研究プロトコール   1. 研究デザイン | （該当する□にチェックをつけてください。）  ☑Ⅰ．臨床研究  ・☑介入研究（介入を伴う研究；下記の①か②を満たすもの）  介入の種類  （☑医薬品、□ワクチン、□遺伝子、□食品、□医療器具・機器、  　□行動・習慣、□手技、□ほか（　　　　　　　　　　））  □①通常の診療を越えた医療行為で、研究が目的  ☑②通常の診療と同等の医療行為で、被験者を２群以上にグループ割付をして、治療、診断、予防などの効果を群間比較する研究）  （グループ割付の方法：☑無作為化　□非無作為化）  ⇒研究計画を指定されたデータベースに事前登録する必要があります。  ・□観察研究（介入を伴わない研究）  （通常の診療範囲内で、被験者の割り付けを伴わない、記録、結果、医療行為に用いた検体等を利用する研究。事例研究など。）  □事例（症例）研究　□予後等追跡調査  □疾病（症例）登録データベース　□症例対照研究  □その他（　　　　　　　　　　　　　　　　　　　　　　　）  ⇒事前に登録する必要はありません。  □Ⅱ．臨床研究ではない研究  （具体的に記述してください。） |
| 1. 主要評価項目   （エンドポイント） | 1. 術後1日目の麻酔覚醒の質 2. 術後1ヶ月および3ヶ月のQOL |
| （３）解析方法 | - 1. 1に関しては翌日の術後診察の際に麻酔覚醒の質に関する患者さんへのアンケート（QoR-40）に答えていただく。   2. 2に関しては泌尿器科外来来院時に麻酔科にも寄っていただく、もしくは自宅に郵送し、QOLに関する患者さんへのアンケート（SF-36）に答えていただく。 |
| （４）共同研究施設の有無 | ☑無  □有（多施設共同研究に該当　□しない　□する　）  ・共同研究施設がある場合は、利用する試料・資料の他施設への提供および他施設からの収集の方法について記載すること。  ・多施設共同研究の場合には、中央事務局による全体の研究計画と共同研究参加施設の一括事前登録をデータベースに行っているかどうかについても記載すること。） |
| ９　利用する試料・資料 | 利用する試料・資料（データ・情報）  □人体から採取された試料（（血液、組織、細胞、排泄物、尿、ほか）  　□新たに採取する（採取方法：　　　　　　　　　　　　　）  □既存試料を利用する  ☑診療情報、□レセプト情報、☑アンケート調査票、□ほか  　☑新たに情報収集する  　□既存資料を利用する |
| 10　個人情報の取り扱い | ☑匿名化する  ☑連結可能匿名化  （連結可能とする情報の管理方法；ログイン時にパスワードが必要なPCで対応する）  □連結不可能匿名化  □匿名化しない |
| 11　研究期間  （研究は原則として、倫理委員会の翌月１日から開始可能。至急開始する必要がある場合は、倫理委員会の翌日以降に開始可能） | 平成23年12月1日～平成26年12月31日 |
| 12　研究にかかる費用  （研究費等の明確化） | ☑奨学寄附金　□受託研究費　□基礎研究費  □科学研究費　□厚生労働科学研究費　□その他  名称（ |
| 13　遵守すべき倫理指針 | ☑臨床研究倫理指針　□遺伝子治療臨床研究倫理指針  □疫学研究倫理指針　□ヒトゲノム・遺伝子解析研究倫理指針  □ヒト幹細胞臨床研究指針　□動物実験実施基本指針  □そのほか（　　　　　　　　　　　　　　　　　　　　　　　　） |
| 14　その他の特記事項 | （１）安全性の確保  　2つの術前補水方法はいずれも一般的に行われてきており、安全性の問題は一般臨床診療のそれを超えない。欧米ではすでにガイドラインとして認められている方法である。  （２）副作用等に対する配慮  　上記の通り、副作用が出ることは考えにくいが、予想される副作用としては、高血糖と誤嚥性肺炎がある。起こった場合は適切に対応する。誤嚥性肺炎に関しては、術前飲水許容による安全性を本学でも2000例を対象に調査し、安全であるとの結論を得ている。  （３）将来の疾病予防・新しい治療方法　等への貢献  いずれの群の術後回復の質が良いかを比較することにより、最良の術前飲水方針を決定する証拠を形成でき、患者予後の向上に貢献できる。 |
