# Supplementary material for: The Effect of Preoperative Oral Carbohydrate or Oral Rehydration Solution on Postoperative Quality of Recovery: A Randomized, Controlled Clinical Trial
Source: PLoS One. 2015 Aug 28;10(8):e0133309. doi: 10.1371/journal.pone.0133309 (PMC4552663; doi:10.1371/journal.pone.0133309)
Supplement: S2 Protocol — (DOCX) [file pone.0133309.s003.docx]

【Revised on February 1^st^, 2011】

Style２

August 8^th^, 2011

Clinical Trial Protocol

【Person in Charge of the Study】Department of Anesthesiology, Ayako Asakura

| １ Title of the Study | Evaluation of Postoperative Quality of Recovery and QOL among Preoperative Fasting, Administration of Oral Carbohydrate, or Oral Rehydration Solution. |
| --- | --- |
| ２ Person in Charge of the Study | Affiliation Department of Anesthesiology  Title, Name Research associate, Ayako Asakura |
| ３ Allotment Researcher | Affiliation Dept. of Anesthesiology Title Professor & Chairperson Takahisa Goto  Operation Dept. Associate professor Tetsuya Miyashita  Dept. of Anesthesiology Assistant Professor Itaru Watanabe  Dept. of Anesthesiology Assistant Professor Gaku Inagawa  Dept. of Anesthesiology Research associate Tatsuya Kondo  Dept. of Anesthesiology Research associate Hiromasa Kawakami  Dept. of Anesthesiology Research associate Hitoshi Sato  Dept. of Anesthesiology Research associate Takayuki Kariya  Dept. of Anesthesiology Research associate Yoshikazu Yamaguchi  Dept. of Anesthesiology Research associate Masashi Yokose  Dept. of Anesthesiology Research associate Akihiro Terahata  Dept. of Anesthesiology Research associate Sayaka Tsuboi  Dept. of Anesthesiology Senior Resident Kyoko Natsukawa  Dept. of Anesthesiology Senior Resident Oki Sato  Dept. of Anesthesiology Senior Resident Atsushi Sakota |
| ４ Personal Information Manager | Affiliation Dept. of Anesthesiology  Title Lecturer, Name Yusuke Mizuno |
| ５ Background and Significance of the Study  (Process which led to this study and expected outcome) | Enhanced postoperative recovery programs, which allow the unrestricted intake of clear liquids until 2 h before anesthesia, have become widely accepted, and preoperative administration of oral carbohydrate or oral rehydration solution has been recommended. Preoperative administration of oral carbohydrate has been shown to reduce preoperative discomfort. Therefore, we expect the higher quality of recovery in preoperative drinking groups than the fasting group. |
| ６ Objective | To evaluate the postoperative quality of recovery and QOL among overnight fasting, preoperative administration of oral carbohydrate or oral rehydration in a transperineal prostate brachytherapy or lymphaticovenular anastomosis. |
| ７ Subjects | Subjects（include sex, age, healthy or patient with specific disease, eligible and exclusion criteria）  ASA physical status 1 and 2, age 20 to 79 years, who are scheduled to undergo a transperineal prostate brachytherapy or lymphaticovenular anastomosis.  Patients with impaired gastrointestinal motility, gastroesophageal reflux, potential difficult airway, poor comprehension of Japanese, or with psychiatric disorders are excluded.  Target number of subjects（Basis of calculation）  250 patients.  Preoperative administration of oral carbohydrate has been shown to reduce preoperative discomfort significantly compared to overnight fasting in total of 250 patients (Anesthesia and Analgesia 2001) |
| ８ Study Protocol   1. Study Design | （Mark the applying box.）  ☑Ⅰ．Clinical Study  ・☑Interventional Study（study which accompany the intervention；those that meet ①or② stated below）  Type of intervention  （☑drugs □vaccine □gene □foods □equipment, devices  　□behavior □procedure □other（　　　　　　　　　　））  □①Medical act beyond the ordinary treatment for the study  ☑②Medical act within the ordinary treatment  Parallel-group trial allocating the subjects to more than two groups comparing the treatment, diagnosis, or prevention, etc.  （Allocation Method：☑Randomized　□Not randomized）  ⇒Need to pre-register the protocol to the specific data base.  ・□Observational Study（study which does not involve the intervention）  （Medical act within the ordinary treatment  Study with no allocation needed, using records, results, or samples from a clinical work. For example, a case study.）  □Case Study　□Follow-up Survey for Prognosis  □Data-base for the Specific Disesase　□Case-Control Study  □Other（　　　　　　　　　　　　　　　　　　　　　　　）  ⇒No need to register  □Ⅱ．Study which is not a clinical study  （Describe specifically.） |
| 1. Primary Outcome   (Endpoint) | 1. Quality of Recovery of the postoperative day 1 2. QOL of the postoperative 1 and 3 months |
| （３）Methods | - 1. Complete the Japanese version of QoR-40 questionnaire on postoperative day 1   2. Complete the SF-36 questionnaire by coming to the hospital or by sending, |
| （４）Collaborative study with other facilities | ☑No  □Yes（Multicenter study　□No　□Yes　）  ・For collaborative study with other facility, describe the way to provide the samples/materials to the other facility or to collect them from there.  ・For multicenter study, state if the whole protocol and collaborative facilities have been pre-register to the specific data base. |
| ９ Samples / Materials | Samples or materials to use（data/ information）  □Samples from human body（blood, tissue, cell, excretion, urine, etc）  　□Gather new samples（method：　　　　　　　　　　　　　）  □Use preexisting samples  ☑Medical information □Receipt information ☑Questionnaire  □Other  　☑Gather new information  　□Use preexisting information |
| 10 Management of Personal Information | ☑Anonymous  ☑Linkable Anonymizing  （Management；use a password required PC）  □Non-linkable anonymizing  □Not anonymous |
| 11 Study Period  （As a genral rule, the study can be started from the next 1^st^ day of the Ethics Committee. If the study needs to be started immediately, it can be started from the next day.） | December 1^st^, 2011～December 31^st^, 2014 |
| 12 Expenses for the Study  (Clarification of the Research Funding) | ☑Endowments for Research □Consigned Research Fund  □Basic Research Fund  □Grant-in-aid for Scientific Research  □Health and Labor Scientific Research Grant  □Other Name（　　　　　　　　　　　　　　　　） |
| 13 Ethic Guidance to follow | ☑Ethical Guidelines for Clinical Study  □Ethical Guidelines for Clinical Research of Gene Therapy  □Ethical Guidelines for Epidemiologic Study  □Ethical Guidelines for Human Genome and Genetic Sequencing Research  □Guidelines for Clinical Study Using Human Stem Cell  □Guidelines for Proper Conduct of Animal Experiment  □Other（　　　　　　　　　　　　　　　　　　　　　　　　） |
| 14 Other Special Notations | （１）Safety ensuring  Both preoperative administration of oral carbohydrate and oral rehydration solution have been accepted as standard practice, and are established in guidelines in the US and Europe.  （２）Consideration for side effects  As mentioned above, it is unlikely that side effects occur, but there is a risk for hyperglycemia and aspiration pneumonia. In case any side effects occurred, it would be treated appropriately. As for aspiration pneumonia, we have followed up 2000 patients who had unrestricted intake of clear liquids till 2 h before anesthesia in our facility, and have concluded the no aspiration pneumonia occurred.  （３）Contribution to preventing a future disease, new therapeutic methods, etc.  If we clarify which solution have best postoperative recovery, we may contribute to a better recovery for a large number of  patients. |
