# Supplementary material for: The Effect of Preoperative Oral Carbohydrate or Oral Rehydration Solution on Postoperative Quality of Recovery: A Randomized, Controlled Clinical Trial
Source: PLoS One. 2015 Aug 28;10(8):e0133309. doi: 10.1371/journal.pone.0133309 (PMC4552663; doi:10.1371/journal.pone.0133309)
Supplement: S1 Supporting Information — (DOC) [file pone.0133309.s004.doc]

様式第１号

平成　23　年　10　月　19　日

臨床研究倫理審議申請書

横浜市立大学附属病院長

　研究責任者

所属　　麻酔科

　　職・氏名　 助教　　　　 朝倉　彩子　　　㊞

　　　　　　　　　　　　　　 　総括責任者

　　　　　　　　　　　　　　　　　　　　　所属　　麻酔科

職・氏名 教授 後藤　隆久　　 ㊞

次の開発・研究を実施したいので、 研究計画書を添えて倫理委員会の審議を申請いたします。

| １　開発・研究名  術前禁飲水、術前経口補水、術前炭水化物補水における麻酔覚醒の質およびQOLの比較 | |
| --- | --- |
| ２　研究責任者 所属　麻酔科 職名 　助教　 　　氏名　朝倉　彩子 | |
| ３　分担研究者 所属 　麻酔科 職名 　教授 　　 　氏名　後藤　隆久  　　　　　　　　　　　　　　　 手術部　　　　　　准教授　　　　　　　　　　宮下　徹也  麻酔科 講師 渡邊　至  麻酔科 講師　　　　　　　　　 伊奈川　岳  麻酔科 助教 近藤　竜也  麻酔科 助教 川上　裕理  麻酔科 助教 佐藤　仁  　　　　　　　　　　　　 麻酔科　　　　　 助教　　　　　　　　　 刈谷　隆之  麻酔科 助教 山口　嘉一  麻酔科 助教 横瀬　真志  麻酔科 助教 寺端　昭博  麻酔科 助教 坪井　さやか  麻酔科 指導診療医 夏川　恭子  　　　　　　　　　　 　麻酔科 　　　 　指導診療医 　 　　佐藤　大樹  　　　　　　　　　　　　　　　 麻酔科　　　　　　 指導診療医　　　　　　　 迫田　厚志 | |
| ４　個人情報管理者　＊研究責任者および分担研究者はなることが出来ない。  　　　　　　　　　　　　　麻酔科　　　　　　講師　　　　　　　　　　水野　祐介 | |
| ５　開発及び研究の概要  近年、手術患者の回復力強化のために、麻酔導入2時間前まで飲水可能とし、経口補水液もしくは炭水化物含有飲料水を摂取することが推奨されてきている。術前に経口補水することで、周術期不快感が減少されることが認められている。  そこで本研究では、小線源埋め込み術およびリンパ管静脈吻合術において、従来通りの術前禁飲水群、術前経口補水群、術前炭水化物補水群の3群で術後の麻酔回復の質、術後1ヶ月および3ヶ月のQOLを調査する。 | |
| ６　開発及び研究の対象（症例数及び対象年齢を含む）並びに実施場所  小線源埋め込み術およびリンパ管静脈吻合術が予定されている20-79歳のASA PS1, 2の患者250人 | |
| ７　実施期間（最長５年） | 平成２３年１２月１日　～　平成２５年１２月３１日 |
| ８　検体、試料、データ等の  保存・管理方法 | データは研究責任者が一括管理する。 |
| ９　開発及び研究における倫理的、社会的配慮について   1. 開発及び研究の対象となる個人の人権の擁護   本研究で得られたデータは匿名化して管理を行い、患者様に社会的不利益が生じないよう配慮する。  調査用紙はシュレッダーにて破棄する。  (２)開発及び研究の対象となる個人に理解を求め同意を得る方法とその範囲（開発・研究名  　　　　を記載して、説明文と同意書を添付する。）  同意書にて本人より同意を得る。  同意の撤回は口頭で可能とする。  (３)開発及び研究の対象となる個人への不利益及び危険性への配慮  　　副作用が出ることは考えにくいが、予想される副作用としては高血糖と誤嚥性肺炎があり、起こった場合には適切に対応する。  (４)医学上の貢献の予測  いずれの群の術後回復の質が良いかを比較することにより、最良の術前飲水方針を決定する証拠を形成でき、患者予後の向上に貢献できる。  (５)その他  （協力病院との共同研究に際しては、協力病院の同意書（案）を添付する。）  協力病院はありません。 | |
