# Supplementary material for: The Effect of Preoperative Oral Carbohydrate or Oral Rehydration Solution on Postoperative Quality of Recovery: A Randomized, Controlled Clinical Trial
Source: PLoS One. 2015 Aug 28;10(8):e0133309. doi: 10.1371/journal.pone.0133309 (PMC4552663; doi:10.1371/journal.pone.0133309)
Supplement: S2 Supporting Information — (DOCX) [file pone.0133309.s005.docx]

Style 1

October 19^th^, 2011

Application for the Ethics Deliberation of Clinical Trials

Director of the Yokohama City University Hospital

Person in Charge of the Study

Affiliation Department of Anesthesiology

Title, Name Research associate, Ayako Asakura

Principle Investigator

Affiliation Department of Anesthesiology

Title, Name Professor & Chairperson, Takahisa Goto

We would like to carry out the following clinical trial.

Therefore, we would like the ethics committee to review our protocol.

| １ Title of the Study  Evaluation of Postoperative Quality of Recovery and QOL among Preoperative Fasting, Administration of Oral Carbohydrate, or Oral Rehydration Solution. | |
| --- | --- |
| ２ Person in Charge of the Study Affiliation Department of Anesthesiology  Title Research associate Name Ayako Asakura | |
| ３ Allotment Researcher Affiliation Dept. of Anesthesiology Title Professor & Chairperson Takahisa Goto  Operation Dept. Associate Professor Tetsuya Miyashita  Dept. of Anesthesiology Assistant Professor Itaru Watanabe  Dept. of Anesthesiology Assistant Professor Gaku Inagawa  Dept. of Anesthesiology Research associate Tatsuya Kondo  Dept. of Anesthesiology Research associate Hiromasa Kawakami  Dept. of Anesthesiology Research associate Hitoshi Sato  Dept. of Anesthesiology Research associate Takayuki Kariya  Dept. of Anesthesiology Research associate Yoshikazu Yamaguchi  Dept. of Anesthesiology Research associate Masashi Yokose  Dept. of Anesthesiology Research associate Akihiro Terahata  Dept. of Anesthesiology Research associate Sayaka Tsuboi  Dept. of Anesthesiology Senior Resident Kyoko Natsukawa  Dept. of Anesthesiology Senior Resident Oki Sato  Dept. of Anesthesiology Senior Resident Atsushi Sakota | |
| ４ Personal Information Manager ＊who is not in charge of the study or not allotment researcher  Dept. of Anesthesiology Lecturer Yusuke Mizuno | |
| ５ Summary of the Study  Enhanced postoperative recovery programs, which allow the unrestricted intake of clear liquids until 2 h before anesthesia, have become widely accepted, and preoperative administration of oral carbohydrate or oral rehydration solution has been recommended. Preoperative administration of oral carbohydrate has been shown to reduce preoperative discomfort. Consequently, the purpose of the current study was to evaluate the postoperative quality of recovery and QOL among overnight fasting, preoperative administration of oral carbohydrate or oral rehydration in a transperineal prostate brachytherapy or lymphaticovenular anastomosis. | |
| ６ Subjects（include target number and age）  250 patients, who are ASA physical status 1 and 2, age 20 to 79 years, and are scheduled to undergo a transperineal prostate brachytherapy or lymphaticovenular anastomosis | |
| ７ Study Period (up to 5 years) | December 1^st^, 2011～December 31^st^, 2014 |
| 8 Preservation and Management of Samples and Data | Person in charge of the study will manage. |
| ９ Social and ethical consideration in the study   1. Protection of human rights for the study subjects     The data will be anonymized, and we will consider that no social disadvantage will occur to the  patients.  We will shred the documents afterward.   1. How to obtain an informed consent from the study subjects（With a study title written down,   submit the explanation and consent together.）  We will obtain the patient’s consent in a written consent.  The withdrawal of the agreement will be possible orally.  (３)Consideration for the safety in the study subjects  It is unlikely that side effects occur, but there is a few risk for hyperglycemia and  aspiration pneumonia. In case any side effects occurred, it would be treated appropriately.  (４)Assumed medical contribution  If we clarify which solution have best postoperative recovery, we may contribute to a  better recovery for a large number of patients.  (５) Other  （If the trial is a collaborative study with other facilities, submit the consent together.）  No other facilities involved. | |
